# Phase I/II study of single-agent lenvatinib in children and adolescents with refractory or relapsed solid malignancies and young adults with osteosarcoma (ITCC-050)^☆^

**DOI:** 10.1016/j.esmoop.2021.100250

**Published:** 2021-09-22

**Authors:** N. Gaspar, Q. Campbell-Hewson, S. Gallego Melcon, F. Locatelli, R. Venkatramani, S. Hecker-Nolting, M. Gambart, F. Bautista, E. Thebaud, I. Aerts, B. Morland, C. Rossig, A. Canete Nieto, A. Longhi, C. Lervat, N. Entz-Werle, S.J. Strauss, P. Marec-Berard, C.E. Okpara, C. He, L. Dutta, M. Casanova

**Affiliations:** 1 Department of Childhood and Adolescent Oncology, Gustave Roussy Cancer Campus, Villejuif, France; 2 The Great North Children's Hospital, Royal Victoria Infirmary, Newcastle Upon Tyne, UK; 3 Pediatric Oncology and Hematology Service, University Hospital Vall d'Hebron, Barcelona, Spain; 4 Department of Pediatric Hematology and Oncology, Ospedale Pediatrico Bambino Gesù, University of Rome, Rome, Italy; 5 Department of Pediatrics, Texas Children's Cancer Center, Baylor College of Medicine, Houston, USA; 6 Department of Pediatric Oncology, Hematology, Immunology, Klinikum Stuttgart - Olgahospital, Stuttgart, Germany; 7 Pediatric Hemato-Oncology Unit, CHU Toulouse - Hôpital des Enfants, URCP, Toulouse, France; 8 Paediatric Haematology-Oncology Department, Hospital Infantil Universitario Niño Jesús, Madrid, Spain; 9 Pediatric Oncology-Hematology and Immunology Department, CHU Nantes - Hôpital Mère-Enfant, Nantes, France; 10 SIREDO Oncology Center, Institut Curie, PSL Research University, Paris, France; 11 Department of Paediatric Hematology/Oncology, Birmingham Children's Hospital, Birmingham, UK; 12 Department of Pediatric Hematology and Oncology, University Children's Hospital Muenster, Muenster, Germany; 13 Children's Oncology Unit, Pediatric Service, Hospital Universitario y Politecnico La Fe, Valencia, Spain; 14 Chemotherapy Service, Istituto Ortopedico Rizzoli IRCCS, Bologna, Italy; 15 Pediatric and AYA Oncology Unit, Centre Oscar Lambret Lille, Lille, France; 16 Pediatric Onco-Hematology Unit, Chu Strasbourg-Hôpital Hautepierre, Strasbourg, France; 17 Clinical Research Facility, University College London Hospitals NHS Trust, London, UK; 18 Institute of Pediatric Hematology and Oncology, Centre Léon Bérard, Lyon, France; 19 Clinical Research, Oncology Business Group, Eisai Ltd., Hatfield, UK; 20 Biostatistics, Oncology Business Group, Eisai Inc., Woodcliff Lake, USA; 21 Clinical Research, Oncology Business Group, Eisai Inc., Woodcliff Lake, USA; 22 Pediatric Oncology Unit, Fondazione IRCCS Istituto Nazionale dei Tumori, Milan, Italy

**Keywords:** lenvatinib, osteosarcoma, pediatric, solid tumors, tyrosine kinase inhibitors

## Abstract

**Background:**

We report results from the phase I dose-finding and phase II expansion part of a multicenter, open-label study of single-agent lenvatinib in pediatric and young adult patients with relapsed/refractory solid tumors, including osteosarcoma and radioiodine-refractory differentiated thyroid cancer (RR-DTC) (NCT02432274).

**Patients and methods:**

The primary endpoint of phase I was to determine the recommended phase II dose (RP2D) of lenvatinib in children with relapsed/refractory solid malignant tumors. Phase II primary endpoints were progression-free survival rate at 4 months (PFS-4) for patients with relapsed/refractory osteosarcoma; and objective response rate/best overall response for patients with RR-DTC at the RP2D.

**Results:**

In phase I, 23 patients (median age, 12 years) were enrolled. With lenvatinib 14 mg/m^2^, three dose-limiting toxicities (hypertension, *n* = 2; increased alanine aminotransferase, *n* = 1) were reported, establishing 14 mg/m^2^ as the RP2D. In phase II, 31 patients with osteosarcoma (median age, 15 years) and 1 patient with RR-DTC (age 17 years) were enrolled. For the osteosarcoma cohort, PFS-4 (binomial estimate) was 29.0% [95% confidence interval (CI) 14.2% to 48.0%; full analysis set: *n* = 31], PFS-4 by Kaplan–Meier estimate was 37.8% (95% CI 20.0% to 55.4%; full analysis set) and median PFS was 3.0 months (95% CI 1.8-5.4 months). The objective response rate was 6.7% (95% CI 0.8% to 22.1%). The patient with RR-DTC had a best overall response of partial response. Some 60.8% of patients in phase I and 22.6% of patients in phase II (with osteosarcoma) had treatment-related treatment-emergent adverse events of grade ≥3.

**Conclusions:**

The lenvatinib RP2D was 14 mg/m^2^. Single-agent lenvatinib showed activity in osteosarcoma; however, the null hypothesis could not be rejected. The safety profile was consistent with previous tyrosine kinase inhibitor studies. Lenvatinib is currently being investigated in osteosarcoma in combination with chemotherapy as part of a randomized, controlled trial (NCT04154189), in pediatric solid tumors in combination with everolimus (NCT03245151), and as a single agent in a basket study with enrollment ongoing (NCT04447755).

## Highlights

• The recommended phase II dose of lenvatinib in children with relapsed/refractory solid malignant tumors is 14 mg/m^2^.
• This dose is equivalent to the recommended dose of 24 mg/day for single-agent lenvatinib in adults with DTC.
• Single-agent lenvatinib showed activity of interest in children and young adults with osteosarcoma.
• Based on this initial report, lenvatinib is currently being investigated in combination with chemotherapy in osteosarcoma.

## Introduction

Cancer in children has a low overall incidence (18.6 cases per 100 000 children); however, it is the third leading cause of death in this age group.^1^^,^^2^ Osteosarcoma is the most commonly diagnosed primary bone neoplasm in children and adolescents.^3^ Approximately 80% of patients with osteosarcoma have localized disease at diagnosis, and 20% present with metastatic disease.^4^ As evidenced by 5-year survival rates (range: 60.6% to 68.1% from 1987 to 2002), there has been minimal progress since the mid-1980s.^5^ Therefore, there remains a high unmet need for new, more effective therapies.

Multiple tyrosine kinase signaling pathways have been implicated in the development of solid tumors, including the vascular endothelial growth factor (VEGF), platelet-derived growth factor (PDGF), and fibroblast growth factor (FGF) receptor pathways.^6^^,^^7^ Specifically, in osteosarcoma, expression of PDGF and PDGF receptors (PDGFRs) has been correlated with disease progression, and high levels of PDGF are associated with lower 5-year progression-free survival (PFS).^8^^,^^9^ Moreover, FGF receptors (FGFRs) are overexpressed in osteosarcoma cells and FGFR signaling enhances tumor cell proliferation.^10^

In addition to osteosarcoma, multiple cell signaling pathways, including VEGF^11^ and RET signaling,^12^ are implicated in the pathogenesis of differentiated thyroid cancer (DTC). In the pediatric population, thyroid cancer is a rare neoplasm, accounting for up to 3% of childhood cancers.^13^ A subset of patients with recurring/relapsing DTC become refractory and/or resistant to radioiodine ablation (RR-DTC), a situation in which cytotoxic chemotherapy is often ineffective.^14^^,^^15^

In previous studies, tyrosine kinase inhibitors (TKIs) have shown some activity in thyroid cancer^16^ and osteosarcoma.^17^ Lenvatinib is an oral TKI targeting VEGF receptors (VEGFRs) 1-3, FGFRs 1-4, PDGFR-α, RET, and KIT.^18^ The half-maximal inhibitory concentration (IC_50_) of lenvatinib for VEGFRs is similar to or better than other kinase inhibitors used in osteosarcoma; and lenvatinib targets FGFR1 and PDGFR-α with a high selectivity (Supplementary Table S1, available at https://doi.org/10.1016/j.esmoop.2021.100250).^19^, ^20^, ^21^, ^22^, ^23^, ^24^, ^25^, ^26^ Currently, lenvatinib is approved for the treatment of adults with RR-DTC in the United States, Europe, and several other countries.^27^

The phase I part of this study aimed to determine the single-agent recommended phase II dose (RP2D) of lenvatinib in children and adolescents with refractory or relapsed solid malignant tumors; the phase II part of this study evaluated the antitumor activity and safety of lenvatinib in relapsed/refractory osteosarcoma and RR-DTC.

## Methods

### Study design

**Figure 1:**
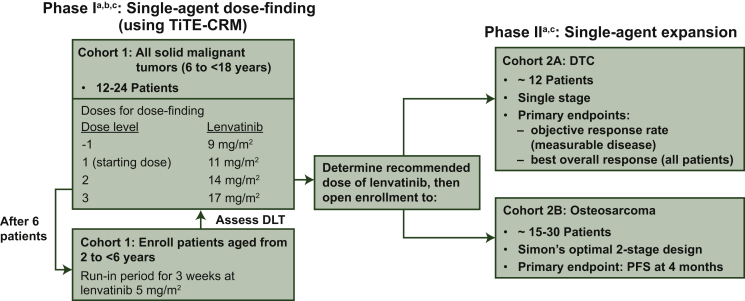
Phase I/II study of lenvatinib in children, adolescents, and young adults (up to 25 years of age with osteosarcoma) with relapsed or refractory solid tumors. BOR, best overall response; DLT, dose-limiting toxicity; DTC, differentiated thyroid cancer; ETP, etoposide; IFM, ifosfamide; LEN, lenvatinib; ORR, objective response rate; PD, progressive disease; PFS-4, progression free survival at 4 months; RD, recommended dose; TiTE-CRM, time to event continual reassessment method. ^a^ The phase I part of this study also included a dose-finding cohort for the combination of lenvatinib and chemotherapy in patients with osteosarcoma (cohort 3A) and the phase II part included an expansion cohort for the combination of lenvatinib and chemotherapy in patients with osteosarcoma (cohort 3B). These cohorts are not shown in this figure, and the results from these cohorts will be published separately. ^b^ Secondary endpoints included safety and toxicity, BOR, ORR, complete response (CR), partial response (PR), duration of response (DOR), PFS, time to progression (TTP), and disease control rate [DCR; CR + PR + durable stable disease (SD) ≥7 weeks]. ^c^ Additional secondary endpoints in phase I/II were identification of blood and tumor biomarkers, population-based pharmacokinetic (PK) parameters and the efficacy of lenvatinib as determined by OS. Exploratory endpoints included assessment of the relationship of lenvatinib exposure to clinical response.

Reprinted from The Lancet Oncology, Vol. 22, Gaspar N, Venkatramani R, Hecker-Nolting S et al. Lenvatinib with etoposide plus ifosfamide in patients with refractory or relapsed osteosarcoma (ITCC-050): a multicentre, open-label, multicohort, phase 1/2 study, Pages 1312 -1321, Copyright 2021, with permission from Elsevier.

This phase I/II multicenter, open-label study (NCT02432274) was conducted at 13 sites in Europe and the United States. The single-agent phase I part of the study included dose-finding cohort 1 and the phase II part included expansion cohorts of patients with RR-DTC (cohort 2A) and osteosarcoma (cohort 2B) treated with lenvatinib monotherapy (Figure 1). The safety and efficacy of lenvatinib monotherapy (cohorts 1, 2A, and 2B) are reported here.

### Study eligibility

Patients in phase I were 2 to <18 years old with confirmed diagnosis of solid tumor that had progressed during or after standard anticancer therapy. Patients in phase II were 2-25 years old in the relapsed/refractory osteosarcoma group or 2-18 years old in the RR-DTC group. Full eligibility criteria for this phase I/II study are included in the Supplementary Methods, available at https://doi.org/10.1016/j.esmoop.2021.100250. The study was conducted in accordance with the International Conference on Harmonisation, Good Clinical Practice (GCP) guidelines, and all applicable local GCP guidelines and regulations. The study protocol, informed consent form, and any related documents were approved by an Institutional Review Board. All patients and/or their legal guardians provided written and signed informed consent and/or assent when applicable. A Protocol Steering Committee provided study oversight after all approvals were obtained.

### Study drug administration

Lenvatinib was administered orally once daily on days 1 to 28 of each 28-day cycle. In phase I, patients ≥6 years of age were assigned to lenvatinib dose levels in accordance with the rules of the Time-to-Event Continual Reassessment Method (TiTE-CRM), which allowed continuous accrual with no trial suspensions. Patients <6 years of age had a run-in period during which they received lenvatinib 5 mg/m^2^/day for 21 days and were assessed for dose-limiting toxicities (DLTs). Patients experiencing a DLT during the run-in period were discontinued from the study; all other patients entered phase I cycle 1. The starting dose of lenvatinib was 11 mg/m^2^/day [∼80% of the adult recommended dose of 24 mg/day adjusted for the standard adult body surface area (BSA) of 1.73 m^2^]. Lenvatinib dosage was capped at 24 mg/day after BSA adjustment in both phase I and phase II. The RP2D in phase I was used in phase II.

### Endpoints

The primary endpoint of phase I was to determine the RP2D of lenvatinib in children and adolescents with relapsed/refractory solid malignancies. DLTs were assessed according to Common Terminology Criteria for Adverse Events (CTCAE) version 4.03. Secondary endpoints of phase I are described in Figure 1.

The phase II primary endpoint was PFS rate at 4 months (PFS-4) for patients with osteosarcoma, and objective response rate (ORR; for patients with measurable disease) and best overall response (BOR) for patients with RR-DTC. The PFS-4 rate was defined as the percentage of patients without progressive disease or initiating new anticancer therapy, at or before 16 weeks after the first dose of study drug, based on investigator assessment per Response Evaluation Criteria In Solid Tumors (RECIST) version 1.1. Phase II secondary endpoints included safety and efficacy of lenvatinib as assessed by BOR (osteosarcoma only), ORR (osteosarcoma only), duration of response (DOR; osteosarcoma and measurable DTC), PFS, time to progression (TTP), disease control rate (DCR), and clinical benefit rate. Additional secondary endpoints in phase I/II are described in Figure 1. Tumors were assessed by investigators per RECIST version 1.1 (phases I/II).

### Assessments and statistical analyses

Phase I planned to enroll 12-24 patients using a TiTE-CRM design. DLTs were assessed during cycle 1 in patients enrolled in cohort 1. At least two patients were required to complete one full 28-day cycle or report a DLT during cycle 1 (at the starting dose) before a patient could be treated at the next dose level (dose escalation). Dose levels could not be skipped when escalating. The RP2D was determined either when ∼18 patients had been tested, or when futility was declared, or when 10 patients had been treated at the same dose. The RP2D was defined as the dose with the DLT rate closest to the target rate of 20%.

For the phase II osteosarcoma cohort, the sample size was estimated as 15-30 patients, based on Simon's Optimal 2-Stage Design,^28^ assuming the null hypothesis PFS-4 (H_0_) was ≤25%, the alternative hypothesis PFS-4 (H_1_) was ≥45%, one-sided type I error was 0.1, and power was 80%. During stage I of the cohort, if at least 5 among the first 15 assessable patients were alive and progression-free at 4 months after the date of first dose, then expansion to 27 patients was continued. If, at the end of the second stage for the cohort, at least 10/27 patients (37%) were alive and progression-free at 4 months, the study drug was considered active.

All safety analyses were carried out on all patients who received ≥1 dose of lenvatinib. Adverse events (AEs) were classified using the Medical Dictionary for Regulatory Activities (MedDRA) version 21.1 and assessed by CTCAE version 4.03 grading. Treatment-emergent AEs (TEAEs) were summarized by study cohort and dose level; changes from baseline were summarized using descriptive statistics.

In phase II, PFS-4 was estimated in all enrolled patients (full analysis set, FAS) by the binomial proportion and Kaplan–Meier estimate. PFS, TTP, and overall survival (OS) were analyzed using Kaplan–Meier product-limit estimates. For pharmacokinetic (PK) analyses, plasma concentrations of lenvatinib were pooled and analyzed using a population PK approach.

Additional discussion of methods (i.e. study design, determination of RP2D, and statistical methods) has been provided within the Supplementary Information, available at https://doi.org/10.1016/j.esmoop.2021.100250.

## Results

### Patients

Patients were enrolled from 29 December 2014 to 31 October 2018; the data cut-off date was 31 March 2017 for phase I, and 2 August 2018 and 31 May 2019 for phase II osteosarcoma and DTC patients, respectively.

**Figure 2:**
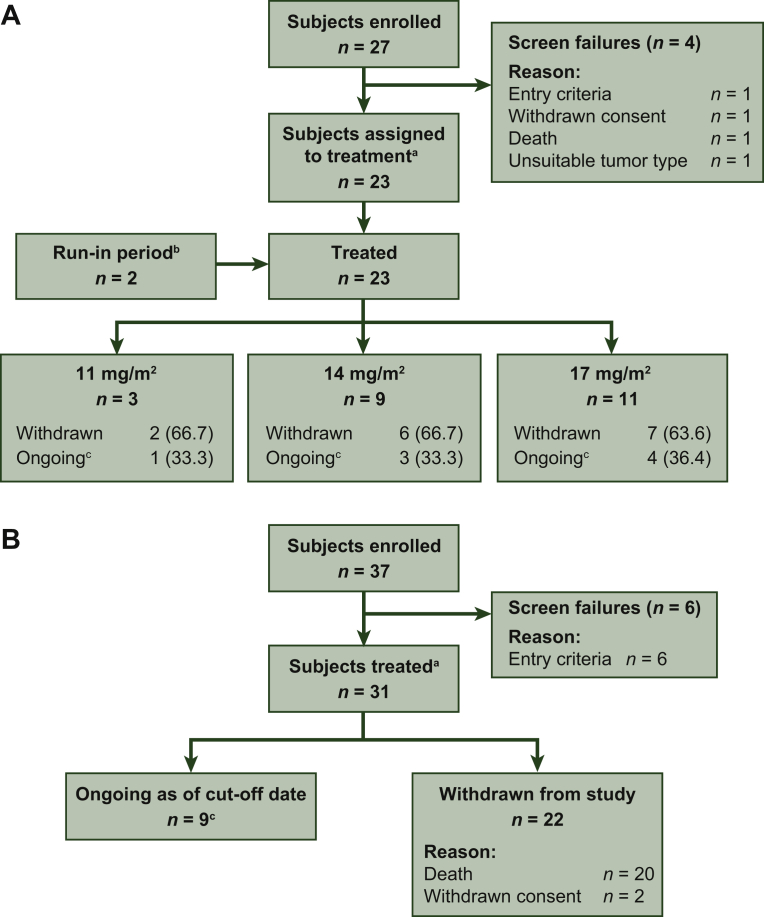
CONSORT diagram for (A) phase I and (B) phase II. ^a^ Patients confirmed to meet eligibility requirements. ^b^ Patients who were 2 to <6 years of age were required to enter a 3-week run-in period, and received lenvatinib 5 mg/m^2^ to confirm tolerability before being assigned to a dose level. Of the two patients who entered the run-in period, one was enrolled at lenvatinib 11 mg/m^2^ and one was enrolled at lenvatinib 14 mg/m^2^. ^c^ Patients who were still receiving study drug or who were in survival follow-up at the cut-off date.

In phase I, 27 patients were screened, with four screening failures (Figure 2A). There were 3 patients in the lenvatinib 11 mg/m^2^, 9 in the 14 mg/m^2^, and 11 in the 17 mg/m^2^ treatment group. Two patients entered the run-in period and joined the main study: one in the lenvatinib 11 mg/m^2^ and one in the 14 mg/m^2^ treatment group. At the phase I cut-off date, one patient with a planned dose of lenvatinib 17 mg/m^2^ was still on treatment (patient was still on treatment at the time of writing this manuscript), whereas all other patients had discontinued the study. The primary reasons for discontinuation were disease progression (11 mg/m^2^ group, *n* = 3; 14 mg/m^2^ group, *n* = 8; 17 mg/m^2^ group, *n* = 8), AE (17 mg/m^2^ group, *n* = 1), patient choice (17 mg/m^2^ group, *n* = 1), and investigator's decision (14 mg/m^2^ group, *n* = 1).

In phase II, 37 patients with osteosarcoma were screened, with six screening failures (Figure 2B). All 31 eligible patients were assigned to the RP2D of lenvatinib 14 mg/m^2^. At the cut-off date (2 August 2018), 3 patients were still on treatment; 28 patients had discontinued treatment. The primary reasons for treatment discontinuation were disease progression (*n* = 25), withdrawal of consent (*n* = 2), and grade 3 arterial thrombosis (*n* = 1). One patient with papillary RR-DTC was enrolled in the study, and stayed on treatment through the data cut-off date (patient was still on treatment at the time of writing this manuscript).

Summaries of patient baseline characteristics are presented in Tables 1 and 2. Patients in phase I had rhabdomyosarcoma (*n* = 5), Ewing sarcoma (*n* = 4), neuroblastoma (*n* = 3), osteosarcoma (*n* = 1), or other solid tumors (brain tumors, *n* = 5; non-brain tumors, *n* = 5). All patients had received prior cytotoxic chemotherapy with the exception of three patients in phase I; two of these three patients underwent a prior anticancer procedure and radiotherapy and the remaining patient had received previous sorafenib treatment. In phase II, all 31 patients with osteosarcoma had prior chemotherapy, 10 patients had prior radiotherapy, and 4 patients had prior anti-VEGF therapy.

**Table 1:** Phase I patient demographics and baseline disease characteristics

Parameter	Phase I lenvatinib dose-finding cohort^a^			
	11 mg/m^2^ (*n* = 3)	14 mg/m^2^ (*n* = 9)	17 mg/m^2^ (*n* = 11)	Total (*N* = 23)
Age, years, median (range)	12 (3-17)	15 (5-17)	12 (6-17)	12 (3-17)
Sex, male, *n* (%)	2 (66.7)	4 (44.4)	6 (54.5)	12 (52.2)
Lansky play score/Karnofsky performance score, *n* (%)^b^				
70	0	1 (11.1)	1 (9.1)	2 (8.7)
80	0	3 (33.3)	3 (27.3)	6 (26.1)
90	0	1 (11.1)	3 (27.3)	4 (17.4)
100	2 (66.7)	3 (33.3)	4 (36.4)	9 (39.1)
Missing^c^	1 (33.3)	1 (11.1)	0	2 (8.7)
Classification of solid tumor type, *n* (%)				
Rhabdomyosarcoma	1 (33.3)	0	4 (36.4)	5 (21.7)
Neuroblastoma	0	0	3 (27.3)	3 (13.0)
Ewing sarcoma	0	3 (33.3)	1 (9.1)	4 (17.4)
Osteosarcoma	1 (33.3)	0	0	1 (4.3)
Other^d^	1 (33.3)	6 (66.7)	3 (27.3)	10 (43.5)
Previous radiotherapy, *n* (%)	2 (66.7)	7 (77.8)	10 (90.9)	19 (82.6)
Previous systemic therapy, *n* (%)	2 (66.7)	8 (88.9)	10 (90.9)	20 (87.0)
Chemotherapy	2 (66.7)	8 (88.9)	10 (90.9)	20 (87.0)
Anthracycline	1 (33.3)	7 (77.8)	8 (72.7)	16 (69.6)
Number of prior systemic anticancer therapies, *n* (%)				
0	1 (33.3)	1 (11.1)	1 (9.1)	3 (13.0)
1	0	2 (22.2)	1 (9.1)	3 (13.0)
2	0	0	4 (36.4)	4 (17.4)
≥3	2 (66.7)	6 (66.7)	5 (45.5)	13 (56.5)
Median Range	3 0-3	3 0-9	3 0-6	3 0-9
Previous anti-VEGF therapy or other tyrosine kinase inhibitor, *n* (%)				
Anti-VEGF therapy^e^	0	2 (22.2)	0	2 (8.7)
Other tyrosine kinase inhibitor (afatinib)	0	1 (11.1)	0	1 (4.3)

Clinical cut-off date: 31 March 2017.

Percentages based on total number of patents within relevant treatment group in the full analysis set.

ECOG PS, Eastern Cooperative Oncology Group performance status; VEGF, vascular endothelial growth factor.

a Due to dose capping, four patients in cohort 1 received a lower dose than planned dose level; two were assigned to the lenvatinib 11 mg/m^2^ group and two were assigned to the lenvatinib 14 mg/m^2^ group. One additional patient was assigned to the lenvatinib 14 mg/m^2^ group due to a dose calculation error.

b Lansky play scores for patients <16 years of age, Karnofsky performance scores for patients ≥16 years of age.

c ECOG PS = 1 for the patient with a planned lenvatinib dose level of 11 mg/m^2^; ECOG PS = 0 for the patient with a planned lenvatinib dose level of 14 mg/m^2^.

d Includes atypical teratoid rhabdoid tumor (*n* = 1), atypical teratoid rhabdoid tumor-like (*n* = 1), alveolar soft part sarcoma (*n* = 1), anaplastic ependymoma (*n* = 1), epithelioid sarcoma (*n* = 1), high-grade glioma (*n* = 1), high-grade undifferentiated soft tissue sarcoma (*n* = 1), medulloblastoma (*n* = 1), papillary thyroid carcinoma (*n* = 1), and paraganglioma (*n* = 1).

e Bevacizumab (*n* = 1), sorafenib (*n* = 1).

**Table 2:** Patient demographics and baseline disease characteristics for phase II patients with osteosarcoma

Parameter	Phase II expansion cohort^a^ Lenvatinib 14 mg/m^2^ (*N* = 31)
Age, years, median (range)	15 (9-22)
Sex, male, *n* (%)	13 (41.9)
Lansky play score/Karnofsky performance score, *n* (%)^b^	
60	1 (3.2)
70	3 (9.7)
80	5 (16.1)
90	15 (32.3)
100	7 (22.6)
Site of lesion, *n* (%)	
Bone only	1 (3.2)
Lung only	15 (48.4)
Lung and bone	14 (45.2)
Prior radiotherapy, *n* (%)^c^	10 (32.3)
For primary tumor	5 (16.1)
For metastases	5 (16.1)
Procedures before study entry, *n* (%)	29 (93.5)
Resection	28 (90.3)
Ablation	3 (9.7)
Biopsy	9 (29.0)
Number of prior systemic anticancer therapies, *n* (%)	
1	7 (22.6)
2	15 (48.4)
≥3	9 (29.0)
Median Range	2 1-6
Previous systemic anticancer agents used by >20% of patients, *n* (%)	
Cisplatin	31 (100.0)
Doxorubicin	30 (96.8)
Methotrexate	30 (96.8)
Ifosfamide	27 (87.1)
Etoposide	19 (61.3)
Docetaxel	10 (32.3)
Gemcitabine	11 (35.5)
Mifamurtide	7 (22.6)
Previous anti-VEGF therapy^d^, *n* (%)	4 (12.9)

Clinical cut-off date: 2 August 2018.

Percentages based on total number of patents within relevant treatment group in the full analysis set.

VEGF, vascular endothelial growth factor.

a Due to dose capping, eight patients received a lower dose than the planned dose of lenvatinib 14 mg/m^2^.

b Lansky play scores for patients ≥1 to <16 years of age, Karnofsky performance scores for patients ≥16 years of age.

c Patients received radiotherapy to lungs, abdomen/pelvis, extremities, and skull/spine/thorax/pelvis.

d Bevacizumab (*n* = 2), pazopanib (*n* = 2).

### Safety

#### Phase I outcomes

In phase I, 3/11 patients had a DLT at the lenvatinib dose of 14 mg/m^2^ (2 of these patients had been initially assigned to the 17 mg/m^2^ dose level, but the DLT was experienced at 14 mg/m^2^). Two patients experienced grade 3 or 4 hypertension as a DLT, which resolved in both patients after dose reduction or discontinuation. One patient had increased serum alanine aminotransferase levels that led to interruption of study drug administration; the DLT improved from grade 3 to grade 2. The RP2D was determined to be lenvatinib 14 mg/m^2^.

Overall, in the phase I part of this study, TEAEs led to dose modification of lenvatinib in 18 patients (78.3%). Dose interruptions occurred in 10/23 patients (median number of days: 14, 11 mg/m^2^; 7, 14 mg/m^2^; and 1, 17 mg/m^2^), and dose reductions occurred in 10/23 patients (Table 3). All patients experienced at least one TEAE; 20/23 had grade ≥3 TEAEs, most of which occurred in one or two patients each (Table 3). Treatment-related TEAEs leading to study drug discontinuation occurred in one patient (grade 4 hypertension; 14 mg/m^2^ group). There were five deaths during treatment; all were considered to be because of disease progression and unrelated to lenvatinib.

**Table 3:** Study treatment exposure and summary of treatment-emergent adverse events in phase I patients with solid tumors or phase II patients with osteosarcoma (safety analysis set)

Parameter	Phase I lenvatinib dose-finding cohort^a^			Phase II expansion cohort^b^
	11 mg/m^2^ (*n* = 5)	14 mg/m^2^ (*n* = 11)	17 mg/m^2^ (*n* = 7)	14 mg/m^2^ (*N* = 31)
Median number of cycles received (range)^c^	6 (2-23)	2 (1-12)	2 (2-7)	3 (1-20)
Median duration of treatment, weeks (range)^d^	20.1 (11.0-89.4)	7.9 (2.0-47.7)	8.0 (6.1-28.0)	11.7 (0.7-76.0)
Median percentage of intended dose (range)^e^	82.9 (58.5-100.0)	87.0 (63.3-100.0)	95.5 (76.4-100.0)	96.3 (45.4-102.1)
Number of patients with dose reduction, *n* (%)	2 (40.0)	5 (45.5)	3 (42.9)	9 (29.0)
Cycle of first dose reduction, *n* (%)				
Cycle 1	0	2 (18.2)	0	1 (3.2)
Cycle 2	0	1 (9.1)	1 (14.3)	6 (19.4)
Cycle 3	0	0	0	0
Cycle 4	0	0	0	0
Cycle 5	1 (20.0)	0	1 (14.3)	1 (3.2)
Cycle ≥6	1 (20.0)	2 (18.2)	1 (14.3)	1 (3.2)
Number of patients with dose interruption, *n* (%)	2 (40.0)	5 (45.5)	3 (42.9)	13 (41.9)
Cycle of first dose interruption, *n* (%)				
1	1 (20.0)	2 (18.2)	0	7 (22.6)
2	0	1 (9.1)	1 (14.3)	2 (6.5)
3	0	1 (9.1)	0	0
4	0	0	0	1 (3.2)
5	0	0	1 (14.3)	2 (6.5)
≥6	1 (20.0)	1 (9.1)	0	1 (3.2)
Median treatment interruption, days (range)	14 (10-18)	7 (2-18)	1 (1-4)	9 (1-36)
TEAEs, *n* (%)	5 (100.0)	11 (100.0)	7 (100.0)	29 (93.5)
Treatment-related^f^	5 (100.0)	8 (72.7)	7 (100.0)	28 (90.3)
Grade ≥3 TEAEs, *n* (%)	4 (80.0)	10 (90.9)	6 (85.7)	20 (64.5)
Treatment-related	2 (40.0)	6 (54.5)	6 (85.7)	7 (22.6)
Serious TEAEs, *n* (%)	2 (40.0)	7 (63.6)	5 (71.4)	21 (67.7)
Treatment-related	0	3 (27.3)	3 (42.9)	9 (29.0)
Deaths^g^, *n* (%)	1 (20.0)	3 (27.3)	1 (14.3)	4 (12.9)
Treatment-related	0	0	0	0
TEAEs leading to drug interruption, *n* (%)	3 (60.0)	9 (81.8)	5 (71.4)	18 (58.1)
TEAEs leading to dose reduction, *n* (%)	2 (40.0)	4 (36.4)	3 (42.9)	9 (29.0)
Treatment-related	2 (40.0)	4 (36.4)	3 (42.9)	9 (29.0)
TEAEs leading to drug discontinuation, *n* (%)	0	3 (27.3)	1 (14.3)	4 (12.9)
Treatment-related	0	1 (9.1)^h^	0	1 (3.2)^i^

Clinical cut-off dates: 31 March 2017 (phase I) and 02 August 2018 (phase II).

Percentages are based on total number of patients within the relevant treatment group for the safety analysis set.

For each TEAE row category, a patient with two or more adverse events in that category is counted only once.

Adverse events coded using Medical Dictionary for Regulatory Activities version 21.1 and grade using Common Terminology Criteria for Adverse Events version 4.03.

TEAE, treatment-emergent adverse event.

a Due to dose capping, four patients in cohort 1 received a lower dose than the planned dose level; two were assigned to the lenvatinib 11 mg/m^2^ group and two were assigned to the lenvatinib 14 mg/m^2^ group. One additional patient was assigned to the lenvatinib 14 mg/m^2^ group due to a dose calculation error.

b Due to dose capping, eight patients received a lower dose than the planned dose of lenvatinib 14 mg/m^2^.

c Patients who received at least one dose of lenvatinib in a cycle were counted in that cycle. Patients were counted in each applicable category.

d Duration of treatment (days) defined as date of last dose of study drug minus date of first dose of study drug + 1.

e Percentage of intended dose defined as dose intensity/planned dose level × 100.

f Treatment-related TEAEs include adverse events that were considered by the investigator to be possibly or probably related to study drug or that had a missing causality on the case report form.

g Fatal TEAEs for any cause. Fatal serious adverse events were also reported in total serious adverse events.

h Grade 4 hypertension led to lenvatinib discontinuation.

i Grade 3 thrombosis led to lenvatinib discontinuation.

#### Phase II outcomes

The median duration of treatment in patients with osteosarcoma was 11.7 weeks (range: 0.7-76.0 weeks), and the median number of treatment cycles was 3 (range: 1-20) (Table 3). Median percentage of the intended dose of lenvatinib received was 96.3% (range: 45.4%-102.1%). Among patients with osteosarcoma, 22/31 had dose interruptions (*n* = 13) or dose reductions (*n* = 9); 69.2% of dose interruptions and 77.8% of dose reductions occurred within the first two cycles of treatment in the phase II cohort (Table 3). Median length of dose interruptions was 9.0 days (range: 1.0-36.0 days). Median time to first dose reduction was 44.0 days (range: 25.0-170.0 days). In the patient with RR-DTC, lenvatinib treatment (14 mg/m^2^) was interrupted for 3 days due to grade 3 diarrhea, and then restarted at 11 mg/m^2^.

In patients with osteosarcoma, 93.5% experienced at least one TEAE (Table 3). Grade ≥3 TEAEs occurred in 20/31 patients, and those occurring in ≥2 patients were back pain (*n* = 5) and tumor pain (*n* = 3). The patient with RR-DTC experienced abdominal, back, and neck pain as well as acne, diarrhea, hypertension, hypertriglyceridemia, hyperuricemia, insomnia, pyrexia, and increased thyroglobulin; all were non-serious AEs. Pneumothorax was reported in five patients with osteosarcoma. One patient had grade 1 pneumothorax (day 104), two patients had grade 2 (day 8; day 48), and two patients had grade 3 (days 34 and 61; days 43 and 140). All patients with pneumothorax had at least one risk factor for this complication, including presence of lung metastases at baseline (*n* = 5), prior lung radiotherapy (*n* = 2), and prior pneumothorax (*n* = 1). None of these patients with pneumothorax underwent prior thoracotomy. Of the five patients with pneumothorax, three had lenvatinib dose interruptions, one had a dose reduction, and one patient discontinued lenvatinib.

In patients with osteosarcoma, treatment-related TEAEs leading to study drug discontinuation occurred in one patient (grade 3 arterial thrombosis). The most common treatment-related TEAEs of grade ≥3 among patients with osteosarcoma were hypertension, diarrhea, proteinuria, decreased weight, and abdominal pain (all *n* = 1) (Supplementary Table S2, available at https://doi.org/10.1016/j.esmoop.2021.100250). There were four deaths during treatment; all were because of disease progression and considered unrelated to study drug treatment. The majority of patients experienced no worsening shift from baseline in hematology parameters, and for those patients who had a shift in toxicity grade, most were 1-grade shifts (Supplementary Table S3, available at https://doi.org/10.1016/j.esmoop.2021.100250).

### Efficacy

#### Phase I outcomes

**Figure 3:**
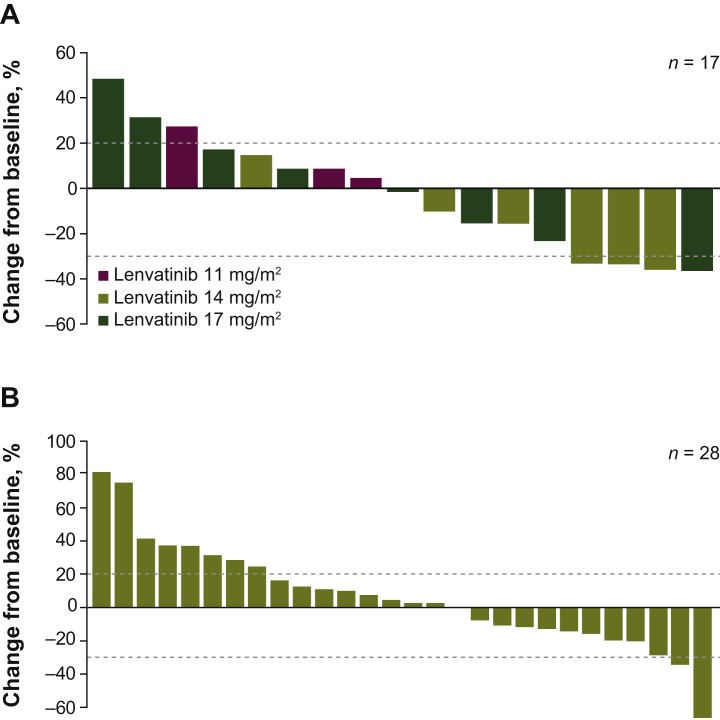
Maximum change in sum of diameters of target lesions in (A) phase I and (B) phase II patients with osteosarcoma.

Among the 22 patients in the phase I cohort with measurable disease, there were no responders. Eleven patients had stable disease (SD), and seven patients with different histologies had durable SD of ≥23 weeks (lenvatinib 14 mg/m^2^, *n* = 3/9; lenvatinib 17 mg/m^2^, *n* = 4/11). The overall median PFS in phase I was 5.0 months [95% confidence interval (CI) 1.6-10.6 months]. Median OS was 7.7 months overall [95% CI 5.5 months to not estimable (NE)]. Tumor shrinkage by dose is reported in Figure 3A.

#### Phase II outcomes

In patients with osteosarcoma, the PFS-4 rate was 29.0% (95% CI 14.2% to 48.0%) based on the FAS (*n* = 31) by binomial estimate (Supplementary Table S4, available at https://doi.org/10.1016/j.esmoop.2021.100250). The Kaplan–Meier estimate of PFS-4 rate was 37.8% (95% CI 20.0% to 55.4%) based on the FAS. Median PFS was 3.0 months (95% CI 1.8-5.4 months) with a median follow-up time of 16.6 months (95% CI 5.5-16.6 months). Two patients experienced partial response (PR), and 13 had SD (Supplementary Table S4, available at https://doi.org/10.1016/j.esmoop.2021.100250). One patient with PR showed a rapid response to lenvatinib treatment, with 34.6% tumor shrinkage at 8 weeks and a DOR of 4.6 months (OS, 11.0 months; PFS, 6.5 months). The second patient with PR experienced 60.0% tumor shrinkage by week 12, with a DOR of 2.8+ months (patient was alive and responding at data cut-off date; OS, 7.9+ months; PFS, 5.5+ months). Tumor shrinkage is shown in Figure 3B.

The ORR in the osteosarcoma cohort was 6.7% (95% CI 0.8% to 22.1%) with a median DOR of 4.6 months (95% CI NE-NE) and median OS of 10.0 months (95% CI 5.6-12.3 months). The DCR was 51.6%, with four patients exhibiting durable SD ≥23 weeks. By the data cut-off date, 74.2% of patients had a progression event. The patient with RR-DTC had a BOR of PR in response to lenvatinib treatment; as of the data cut-off date, the patient had received 185 days (seven cycles) of study drug. The DOR for this patient was 1.9 months, PFS was 5.5 months, and OS was 6.1 months. This patient remains on treatment at the time of development of this report.

Results of the PK analyses in this study (which were comparable to previous studies with adult populations) can be found in the Supplementary Figures S1 and S2, available at https://doi.org/10.1016/j.esmoop.2021.100250.

## Discussion

This phase I/II study demonstrated the safety and preliminary antitumor activity of lenvatinib in children, adolescents, and young adults with relapsed/refractory solid tumors and osteosarcoma. Toxicity in this phase I/II study was consistent with previous studies of lenvatinib.^18^ In phase I, three DLTs occurred in the lenvatinib 14 mg/m^2^ group; this dose was identified as the RP2D. PK analysis indicated that no age-based dose adjustments were required because lenvatinib clearance was not affected by age. Predicted exposure levels based on dosing by BSA at the recommended pediatric dose (14 mg/m^2^ once daily) were comparable to those in adults with DTC (lenvatinib 24 mg once daily). Dose modifications were successfully used to manage lenvatinib toxicity in children, adolescents, and young adults (<25 years old) and the treatment discontinuation rate was low.

In phase I, 61% of patients experienced grade ≥3 treatment-related TEAEs. The most frequently reported were hypothyroidism, diarrhea, vomiting, decreased appetite, and hypertension, consistent with other TKI phase I studies.^29^, ^30^, ^31^ In phase II, lenvatinib showed an acceptable safety profile. The AEs reported were manageable with dose modifications. In the osteosarcoma cohort, all five patients who experienced pneumothorax (all were grade ≤3) had lung metastases. Pneumothorax resulted in dose modifications (interruption/reduction) in four patients and lenvatinib discontinuation in one patient. The incidence of pneumothorax in this study (phase I/II; 11%) is similar to that for other TKIs (3%-16%), and is consistent with the prevalence observed in patients with relapsed/refractory osteosarcoma.^17^^,^^25^^,^^30^^,^^32^, ^33^, ^34^, ^35^, ^36^, ^37^

Our study was limited by the enrollment of only one patient with RR-DTC. Therefore, no conclusions can be drawn about the activity of lenvatinib in RR-DTC. However, it is important to note that this patient with RR-DTC remains on treatment (at the time of this report's development) with a BOR of PR. Another study limitation included the short median follow-up time.

Osteosarcoma trials are increasingly utilizing the PFS-4 rate as a measure of antitumor activity,^25^^,^^38^, ^39^, ^40^ as ORR may be less accurate because of lesion calcification, which can increase lesion size and inaccurately suggest disease progression.^41^^,^^42^ Among patients with osteosarcoma in the phase II cohort, lenvatinib monotherapy demonstrated promising antitumor activity. As such, the Kaplan–Meier estimate of PFS-4 rate (FAS) was 37.8% (95% CI 20.0% to 55.4%), PFS-4 rate was 29.0% per binomial estimate (FAS), and median OS was 10.0 months (95% CI 5.6-12.3 months). These results suggest that single-agent lenvatinib may have activity in osteosarcoma, although the null hypothesis could not be rejected. The efficacy results for lenvatinib in patients with osteosarcoma were in line with those seen with other systemic TKI treatments. Specifically in previous phase II studies of TKI monotherapies, PFS-4 rates ranged from 46% to 79% and median PFS ranged from 3.6 months to 6.7 months (Supplementary Table S5, available at https://doi.org/10.1016/j.esmoop.2021.100250).^17^^,^^25^^,^^30^^,^^37^^,^^38^

In summary, the null hypothesis could not be rejected; however, lenvatinib did show activity of interest in osteosarcoma with a safety profile consistent with that observed for other TKIs and the known safety profile of lenvatinib.^18^^,^^43^^,^^44^ It has been suggested that antiangiogenic TKIs may enhance the effectiveness of conventional chemotherapy regimens.^6^ Consequently, in combination with other agents like chemotherapy, lenvatinib might have a greater efficacy for patients with relapsed/refractory osteosarcoma. As such, lenvatinib in combination with chemotherapy (ifosfamide and etoposide) is currently being investigated (NCT04154189; ITCC-082). Additionally, single-agent lenvatinib is being further investigated in other solid tumors in the HopSkip study (NCT04447755) and in combination with everolimus in the E7080-A001-216 study (NCT03245151).
