# Supplementary material for: Phase I/II study of single-agent lenvatinib in children and adolescents with refractory or relapsed solid malignancies and young adults with osteosarcoma (ITCC-050)☆
Source: ESMO Open. 2021 Sep 22;6(5):100250. doi: 10.1016/j.esmoop.2021.100250 (PMC8477142; doi:10.1016/j.esmoop.2021.100250)
Supplement: Supplementary Material [file mmc1.docx]

**Supplementary Information**

**METHODS**

***Study design***

The phase I part of this study included a dose-finding cohort for the combination of lenvatinib and chemotherapy in patients with osteosarcoma (Cohort 3A). The phase II part included expansion cohorts (**Figure 1**) of patients with RR-DTC (Cohort 2A) and osteosarcoma (Cohort 2B) treated with lenvatinib monotherapy; Cohort 3B included patients with osteosarcoma treated with lenvatinib plus chemotherapy.

***Inclusion and exclusion criteria***

In addition to confirmed diagnosis of relapsed or refractory solid malignant tumors, eligible patients had evaluable or measurable disease per RECIST v1.1, adequate organ function, life expectancy of ≥3 months, Lansky Play score or Karnofsky Performance status score ≥50%, and adequate blood pressure control. Patients with osteosarcoma had to be in relapse.

Patients were excluded if they had any active infections or infectious illness, organ toxicity due to prior anticancer therapy, were currently on any other antitumor therapy, had previously been treated with lenvatinib outside of the current study, had received ≥2 previous VEGF/VEGFR-targeted therapies, were currently or within the 30 days preceding informed consent enrolled in another clinical trial, or for any electrocardiogram abnormality, gastrointestinal malabsorption or bleeding or any active second malignancy within 2 years before enrollment (in addition to the primary tumor types, but not including superficial melanoma, in situ, basal or squamous cell carcinoma of the skin).

***Assessment of dose limiting toxicity***

DLT was assessed according to CTCAE version 4.03 and was defined as grade 4 neutropenia for ≥7 days, grade ≥3 thrombocytopenia with bleeding or lasting >7 days, grade ≥3 febrile neutropenia, grade ≥3 nonhematological toxicity persisting >7 days despite supportive care, grade 4 hypertension, confirmed systolic or diastolic blood pressure more than 25mm Hg above the 95^th^ percentile for age or elevated diastolic blood pressure not controlled by a single antihypertensive medication within 14 days of use, grade 3 proteinuria, any recurrent grade 2 nonhematological toxicity requiring ≥2 dose interruptions or reductions, any dose reductions or interruptions due to toxicity that resulted in administration of <75% of planned dose for lenvatinib, or any other grade ≥3 toxicity assessed as related to lenvatinib treatment, and which in the opinion of the principal investigator and sponsor constitutes a DLT.

***Determination of the RP2D***

Cohort 1 was defined as a lenvatinib single-agent dose-finding study including up to 24 patients. A TiTE-CRM design was used to determine the RP2D of lenvatinib and to increase the flexibility by allowing continuous accrual throughout the study while using the 4-week toxicity endpoint as the basis for dose escalation [Doussau A et al. *Contemp Clin Trials*. 2012;33(4):657-665]. This design allows for continuous accrual with no trial suspensions, which are typically needed when the toxicity assessment of patients previously recruited, is not completed [Smith M et al. *J Clin Oncol*. 1998;16(3):966-978; Cheung Y. *Clin Trials*. 2013;10(6):852-861]. Using this TiTE-CRM design, an eligible patient could be included in the trial at any time, without waiting for the completion of prior patients [Doussau A et al. *Contemp Clin Trials*. 2012;33(4):657-665]. The model was re-estimated considering all the toxicity observations currently available. The newly enrolled patient was treated at the best current estimate of the RP2D. Individual patients on long-term treatment may be treated at a dose below the dose recommended by the model for safety reasons. For patients in Cohort 1, the actual dose level could have been different from the planned dose level due to BSA adjustment and dose capping of lenvatinib at 24 mg. For the purposes of dose-determination in Cohort 1, the Safety Analysis Set was based on the actual dose that each patient received, adjusted for BSA.

The RP2D was defined as the dose that had DLT rate closest to the targeted 20% rate. Four experimental lenvatinib doses were allowed in phase I: 9 mg/m^2^ (dose -1), 11 mg/m^2^ (dose 1), 14 mg/m^2^ (dose 2), and 17 mg/m^2^ (dose 3). The starting dose was 11 mg/m^2^.

A one-parameter empirical power model was used to assess the relationship between the dose level and the probability of DLT: *F(d,α) =* *p*_d_*^exp(α)^*, where F(d,α) is the estimated probability of DLT at dose-level d, *p*_d_ is the prior probability of DLT at dose level d, and α is the unknown parameter to be estimated by the model. The vector {p_0d_} represents the initial guesses of toxicity probabilities, reflecting the clinicians’ prior impression. The skeleton of initial guesses of toxicity probabilities {p_0d_} was numerically calibrated using the approach of [Lee D et al. *J Clin Oncol*. 2005;23(33):8431-8441] and [Cheung et al. *Biometrics*. 2000;56(4):1177-1182], using the “getprior” function of R, ensuring good design operating characteristics. Based on consultation with the clinicians, the delta (half of the width of the CI) defining the indifference interval was set at 0.06 (indifference interval: 0.14 to 0.26) and the prior maximum tolerated dose (MTD_0_) at dose 2, (14 mg/m²) is likely to be the RP2D (same as in adults). This yields a vector of prior probabilities {p_0k_} equal to 0.03, 0.10, 0.20, and 0.33, for the lenvatinib doses 9 mg/m^2^, 11 mg/m^2^, 14 mg/m^2^, and 17 mg/m^2^, respectively, that was found reasonable by the clinicians.

A noninformative prior distribution Normal (0, 1.34) was assigned for α in the Bayesian computation. The simulation study confirmed that the operating characteristics of the model defined with these parameters were reasonable, with more than 50% correct selection of the RP2D in three contrasted scenarios.

Starting with dose 1, the prior distribution of the parameter α was updated by the accruing data on DLTs each time a patient completed evaluation for toxicity in cycle 1. Additional patients were allocated to the dose associated with the posterior probability of DLT closest to the target (ie, having a DLT rate closest to 20%). At least two patients were required to complete one full 28-day cycle or report a DLT during cycle 1 (at the starting dose) before a patient could be treated at the next dose level (dose-escalation). Dose levels could not be skipped when escalating. Intrapatient dose escalation was not allowed for patients 6 years and older; it was only allowed for patients 2 to <6 years old when they started cycle 1. The RP2D was determined either when approximately 18 patients had been tested, or when futility was declared or when 10 patients had been treated at the same dose. Futility was defined as having <25% probability that any of the doses is safe.

***Crossover to cohorts with chemotherapy***

Crossover to cohorts with lenvatinib plus chemotherapy treatment was permitted for patients in this study who progressed on monotherapy and were eligible for chemotherapy; however, no patients crossed over in this study.

***Additional statistical analyses***

PFS-4 rate was estimated using the binomial proportion with corresponding 80% and 95% exact binomial distribution confidence intervals as determined by the Clopper and Pearson method. The PFS-4 rate was tested using the null hypothesis that the PFS-4 rate is ≤25% tested against the alternative hypothesis that the PFS-4 rate was ≥45% using the one-sample exact test of a single proportion at the one-sided 0.1 level. The cumulative probability of PFS at 4 and 12 months was analyzed by Kaplan–Meier product-limit estimates, and presented with 2-sided 95% CI when an adequate number of at-risk patients warranted the estimate.

**RESULTS**

***Pharmacokinetic (PK) Results***

Mean and median plasma concentrations of lenvatinib were not linearly dose proportional across the three dose cohorts (**Supplementary Figure S1**), likely because of the effect of body weight on oral clearance (CL/F). Lenvatinib PK modelling showed CL/F increasing in patients with increasing body weight and decreasing in patients with low body weight (which led to an increase in lenvatinib area under the concentration-time curve [AUC]). PK data in this study show a similar effect of body weight on lenvatinib PK parameters, as body weight and body surface area (BSA) were highly correlated with lenvatinib clearance (**Supplementary Figure S2**). Modelled systemic exposures at AUC_ss_ for pediatric patients receiving lenvatinib 14 mg/m^2^ were comparable to adult patients receiving an equivalent dose in Study 303 (data not shown).

**Supplementary Table S1. Inhibition of kinase activity by tyrosine kinase inhibitors targeting cancer**

| **Target** | **IC_50_ (nM)** | | | | |
| --- | --- | --- | --- | --- | --- |
|  | **Sorafenib**  **[Grande 2012; Matsuki 2018]** | **Cabozantinib**  **[Yakes 2011; Roskoski 2016]** | **Apatinib**  **[Tian 2011; Xie 2019]** | **Regorafenib**  **[Wilhelm 2011; Roskoski 2016]** | **Lenvatinib**  **[Matsui 2008, Matsuki 2018, Grande 2012]** |
| VEGFR-1 | 26 | – | 70 | – | 22 |
| VEGFR-2 | 90 | 0.035 | 2 | 3 | 4 |
| VEGFR-3 | 20 | – | – | 135 | 5.2 |
| FGFR1 | – | – | >10,000 | – | 46 |
| PDGFR-α | – | – | 537 | – | 51 |
| c-KIT | 68 | 4.6 | 420 | 22 | 100 |
| RET | 47 | 5.2 | 13 | ~10 | 35 |
| MET | – | 1.3 | – | – | – |
| Binding mode | VEGFR: type II | VEGFR: type I | – | VEGFR: type II | VEGFR and FGFR: type V |

FGFR, fibroblast growth factor receptor; IC_50_, inhibitory concentration 50; PDGFR, platelet-derived growth factor receptor; VEGFR, vascular endothelial cell growth factor receptor.

**Supplementary Table S2. Most common treatment-related TEAEs in ≥ 10% of patients in phase I patients with solid tumors or phase II patients with osteosarcoma (safety analysis set)**

| **MedDRA preferred term, *n* (%)** | **Phase I lenvatinib dose-finding cohort** | | | | | | **Phase II expansion cohort** | |  |
| --- | --- | --- | --- | --- | --- | --- | --- | --- | --- |
|  | **11 mg/m^2^  (*n* = 5)** | | **14 mg/m^2^  (*n* = 11)** | | **17 mg/m^2^  (*n* = 7)** | | **14 mg/m^2^  (*n* = 31)** | |  |
|  | Any grade | Grade ≥3 | Any grade | Grade ≥3 | Any grade | Grade ≥3 | Any grade | Grade ≥3 | |
| **Patients with treatment-related TEAE, *n* (%)** | 5 (100.0) | 2 (40.0) | 8 (72.7) | 6 (54.5) | 7 (100.0) | 6 (85.7) | 28 (90.3) | 7 (22.6) | |
| **Hypothyroidism^a^** | 3 (60.0) | 0 | 6 (54.5) | 0 | 3 (42.9) | 0 | 13 (41.9) | 0 | |
| **Decreased appetite** | 3 (60.0) | 0 | 5 (45.5) | 1 (9.1) | 1 (14.3) | 0 | 13 (41.9) | 0 | |
| **Hypertension^b,c^** | 2 (40.0) | 0 | 3 (27.3) | 2 (18.2) | 4 (57.1) | 3 (42.9) | 10 (32.3) | 1 (3.2) | |
| **Blood TSH increased^d^** | 0 | 0 | 0 | 0 | 0 | 0 | 9 (29.0) | 0 | |
| **Asthenia** | 2 (40.0) | 0 | 1 (9.1) | 0 | 0 | 0 | 8 (25.8) | 0 | |
| **Fatigue** | 2 (40.0) | 0 | 2 (18.2) | 0 | 3 (42.9) | 1 (14.3) | 8 (25.8) | 0 | |
| **Diarrhea** | 3 (60.0) | 0 | 5 (45.5) | 0 | 3 (42.9) | 0 | 8 (25.8) | 1 (3.2) | |
| **Nausea** | 1 (20.0) | 0 | 2 (18.2) | 0 | 3 (42.9) | 0 | 8 (25.8) | 0 | |
| **Vomiting** | 1 (20.0) | 0 | 6 (54.5) | 0 | 3 (42.9) | 0 | 7 (22.6) | 0 | |
| **Proteinuria^e,f^** | 0 | 0 | 3 (27.3) | 1 (9.1) | 3 (42.9) | 1 (14.3) | 7 (22.6) | 1 (3.2) | |
| **Weight decreased** | 3 (60.0) | 1 (20.0) | 4 (36.4) | 1 (9.1) | 1 (14.3) | 0 | 6 (19.4) | 1 (3.2) | |
| **Abdominal pain** | 2 (40.0) | 0 | 2 (18.2) | 0 | 1 (14.3) | 0 | 5 (16.1) | 1 (3.2) | |
| **Headache** | 0 | 0 | 1 (9.1) | 0 | 1 (14.3) | 0 | 5 (16.1) | 0 | |
| **Dysphonia** | 0 | 0 | 0 | 0 | 0 | 0 | 5 (16.1) | 0 | |
| **Hair color changes** | 1 (20.0) | 0 | 1 (9.1) | 0 | 1 (14.3) | 0 | 2 (6.5) | 0 | |
| **ALT increased** | 0 | 0 | 2 (18.2) | 1 (9.1) | 1 (14.3) | 0 | 1 (3.2) | 0 | |
| **Arthralgia** | 0 | 0 | 2 (18.2) | 0 | 1 (14.3) | 1 (14.3) | 1 (3.2) | 0 | |
| **Myalgia** | 1 (20.0) | 1 (20.0) | 2 (18.2) | 0 | 0 | 0 | 1 (3.2) | 0 | |
| **Palmar-plantar erythrodysesthesia** | 2 (40.0) | 0 | 2 (18.2) | 0 | 0 | 0 | 1 (3.2) | 0 | |
| **Pain in extremity** | 0 | 0 | 2 (18.2) | 0 | 1 (14.3) | 0 | 0 | 0 | |
| **Erythema** | 1 (20.0) | 0 | 1 (9.1) | 0 | 1 (14.3) | 0 | 0 | 0 | |

Clinical cutoff dates: 31 March 2017 (phase I) and 02 August 2018 (phase II).

Percentages based on total number of patients within the relevant treatment group for the safety analysis set. Adverse events coded using MedDRA version 21.1. Treatment-related TEAEs include adverse events that were considered by the investigator to be possibly or probably related to study drug or that had a missing causality on the case-report form.

ALT, alanine aminotransferase; MedDRA, Medical Dictionary for Drug Regulatory Activities; TEAE, treatment-emergent adverse event; TSH, thyroid-stimulating hormone.

^a^In phase I, all any-grade hypothyroidism adverse events were grade 2. In phase IIb, 10 of 13 any-grade hypothyroidism adverse events were grade 2.

^b^Hypertension occurred in patients with anaplastic epemdymoma, osteosarcoma, papillary thyroid cancer, Ewing sarcoma, atypical teratoid rhabdoid tumor, rhabdomyosarcoma, and neuroblastoma in phase I.

^c^Previous anticancer treatments in patients experiencing hypertension included actinomycines, anti-GD2 monoclonal antibody, busulfan, carboplatin, celecoxib, cisplatin, cyclophosphamide, dactinomycin, docetaxel, doxorubicin, etoposide, gemcitabine, ifosfamide, interleukin-2, iobenguane, irinotecan, melphalan, methotrexate, mifamurtide, temozolomide, thiotepa, topotecan, tretinoin, vincristine, and vinorelbine.

^d^In phase II, seven of nine any-grade blood TSH increased adverse events were grade 2.

^e^Proteinuria occurred in patients with alveolar rhabdomyosarcoma, papillary thyroid cancer, Ewing sarcoma, and atypical teratoid rhabdoid tumor-like in phase I.

^f^Previous anticancer treatments in patients experiencing proteinuria included actinomycines, busulfan, cisplatin, carboplatin, cyclophosphamide, dactinomycin, docetaxel, doxorubicin, etoposide, gemcitabine, ifosfamide, irinotecan, melphalan, methotrexate, mifamurtide, pembrolizumab, temozolomide, topotecan, and vincristine.

**Supplementary Table S3. Shifts in toxicity grade from Grade 0, 1, or 2 at baseline to Grade 3 or 4 postbaseline in key hematology parameters in phase II patients with osteosarcoma**

| **Hematologic Parameter** | **Phase II expansion cohort^a^** | |
| --- | --- | --- |
|  | **Lenvatinib 14 mg/m^2^**  **(n = 31)**  **n (%)** | |
| **Postbaseline Grade** | **Grade 3** | **Grade 4** |
| **Baseline Grade** |  |  |
| Hemoglobin decreased (g/L) |  |  |
| 1 | 1 (3.3) | 0 |
| 2 | 0 | 0 |
| WBC count decreased (10^9^/L) |  |  |
| 2 | 2 (6.7) | 1 (3.3) |
| Lymphocytes decreased (10^9^/L) |  |  |
| 0 | 0 | 0 |
| 1 | 1 (3.3) | 0 |
| 2 | 1 (3.3) | 0 |
| Neutrophils decreased (10^9^/L) |  |  |
| 2 | 1 (3.3) | 1 (3.3) |
| Platelets decreased (10^9^/L) |  |  |
| 0 | 1 (3.3) | 1 (3.3) |
| 1 | 0 | 1 (3.3) |

Clinical cutoff date: 02 August 2018.

Table includes only parameters for which there was a shift to Grade 3 or 4 postbaseline.

Rows containing only zeroes have been excluded.

Grade 0 includes laboratory results that could not be graded using CTCAE version 4.03.

Percentages are based on the number of patients with nonmissing data and postbaseline.

CTCAE, Common Terminology Criteria for Adverse Events; WBC, white blood cell.

^a^Due to dose capping, eight patients received a lower dose level than the planned dose of lenvatinib 14 mg/m^2^.

**Supplementary Table S4. Efficacy outcomes in phase II patients with osteosarcoma based on RECIST v1.1 by investigator assessment (full analysis set)**

|  | **Phase II expansion cohort^a^** |
| --- | --- |
|  | **Lenvatinib 14 mg/m^2^** |
| **Patients in full analysis set, *n*** | 31 |
| **PFS-4 by binomial estimate, *n* (%)** | 9 (29.0) |
| **95% CI** | (14.2─48.0) |
| **Median PFS, months (95% CI)** | 3.0 (1.8─5.4) |
| **PFS rate by Kaplan-Meier estimate, % (95% CI) at:** |  |
| **4 months** | 37.8 (20.0─55.4) |
| **12 months** | 5.9 (0.4─22.9) |
| **Median follow-up time for PFS, months (95% CI)** | 16.6 (5.5–16.6) |
| **Median OS, months (95% CI)** | 10.0 (5.6–12.3) |
| **Patients with measurable disease, *n* (%)** | 30 (96.8) |
| **Best overall response, *n* (%)** |  |
| **PR** | 2 (6.7) |
| **SD** | 13 (43.3) |
| **PD** | 12 (40.0) |
| **Not evaluable^b^** | 3 (10.0) |
| **Objective response rate, CR + PR, *n* (%)** | 2 (6.7) |
| **95% CI** | (0.8–22.1) |
| **Median duration of objective response, months (95% CI)** | 4.6 (NE–NE) |
| **Disease control rate (CR + PR + SD ≥7 weeks), *n* (%)** | 16 (51.6) |
| **95% CI** | 33.1–69.8 |

Clinical cutoff date: 02 August 2018.

Percentages are based on total number of patients within the relevant treatment group in the full analysis set.

Rows containing only zeroes have been omitted from the table.

CI, confidence interval; CR, complete response; FAS, full analysis set; NE, not estimable; OS, overall survival; PD, progressive disease; PFS, progression-free survival; PFS-4, progression-free survival at 4 months; PR, partial response; RECIST v1.1, Response Evaluation Criteria In Solid Tumors version 1.1; SD, stable disease.

^a^Due to dose capping, eight patients received a lower dose level than the planned dose of lenvatinib 14 mg/m^2^.

^b^Not evaluable means best overall response of not evaluable or SD of <7 weeks duration posttreatment.

**Supplementary Table S5. Efficacy results from previous phase II studies of TKI monotherapies in patients with osteosarcoma**

| **Study Drug**  **(reference)** | **Patient Population** | **PFS-4, %**  **(95% CI)** | **Median PFS, months (95% CI)** | **Median OS, months (95% CI)** |
| --- | --- | --- | --- | --- |
| **Sorafenib (Grignani et al 2012)** | Metastatic osteosarcoma  median age: 21 years; range 15–62  *n* = 35 | 46  (28–63) | 4  (2–5) | 7  (7–8) |
| **Regorafenib^a^**  **(Duffaud et al 2019)** | Metastatic osteosarcoma  median age: 33 years; range 22–50  *n* = 38 | NA | 4.1^b^  (2–6.8) | 11.3  (5.9–23.9) |
| **Regorafenib**  **(Davis et al 2019)** | Metastatic osteosarcoma  median age: 37 years; range 18–76  *n* = 42 | 79.0 (NA) | 3.6  (2.0–7.6) | 11.1  (4.7–26.7) |
| **Cabozantinib**^c^  **(Italiano et al 2020)** | Advanced osteosarcoma  Median age: 34 years: range 20–53  *n* = 45 | 71  (55─83) | 6.7  (5.4–7.9) | 10.6  (7.4–12.5) |
| **Apatinib**  **(Xie et al 2019)** | Progressive relapsed or unresectable osteosarcoma  median age 23.4 years; range 16–62  *n* = 37 | 56.8  (39.4–70.8) | 4.5  (3.47–6.27) | 9.87  (7.97–18.93) |

NA, not available.

^a^The proportion of patients with no progression at 6 months was 35% in regorafenib-treated patients.

^b^This data was provided in weeks in Duffaud et al, 2019. It was converted to months for use in this table (4 weeks per month).

^c^The proportion of patients with no progression at 6 months was 26% in cabozantinib-treated patients; PFS was 33% (95% CI 19–48) at 6 months.

**Supplementary Figure S1A–D. Lenvatinib plasma concentration by dose level and time point in phase I and phase II.** (A) Phase 1, 11 mg/m^2^ group; (B) Phase 1, 14 mg/m^2^ group; (C) Phase 1, 17 mg/m^2^ group; (D) Phase 2.


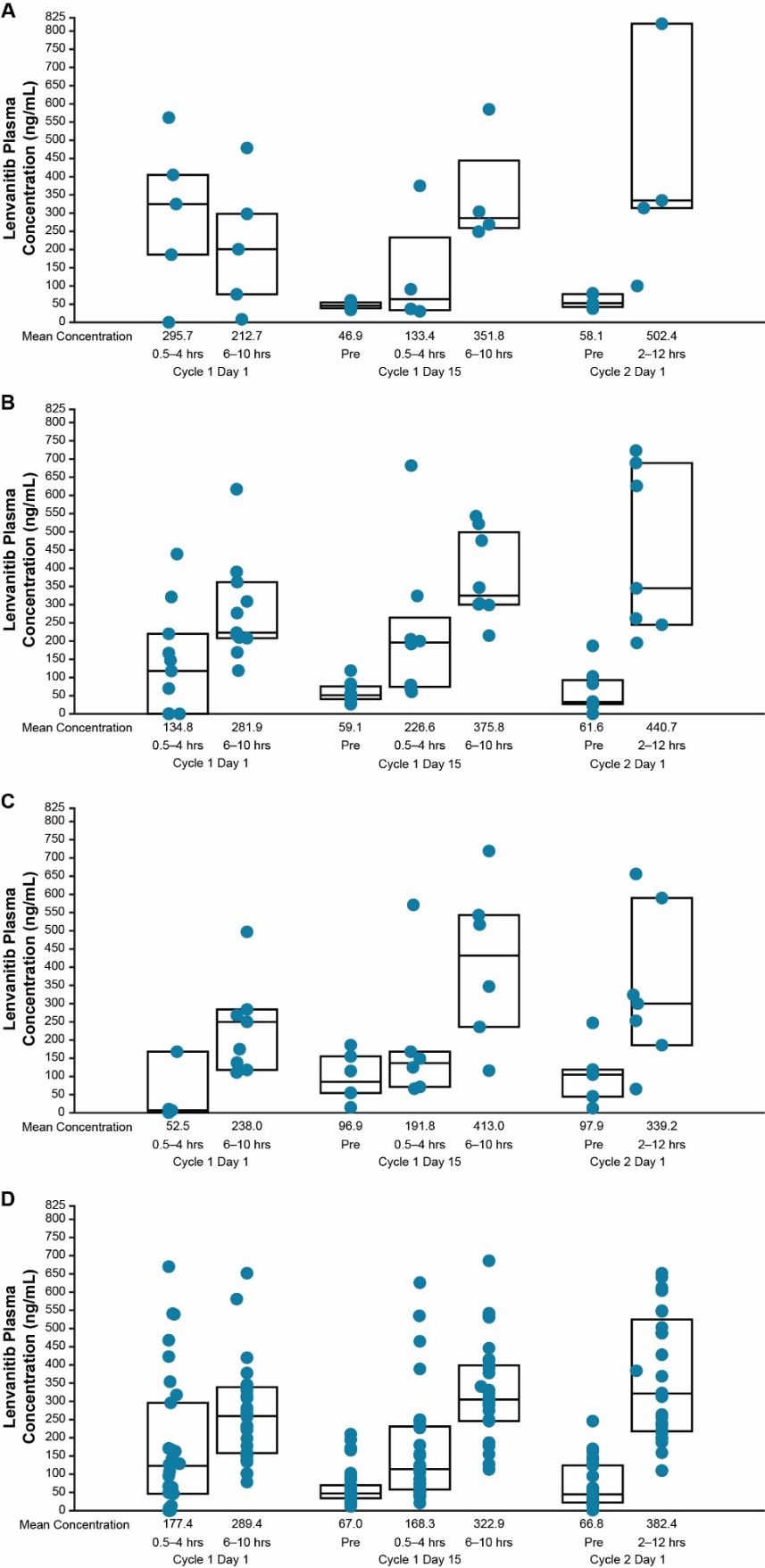


**Supplementary Figure S2. Lenvatinib oral clearance is correlated with body weight and body surface area.**


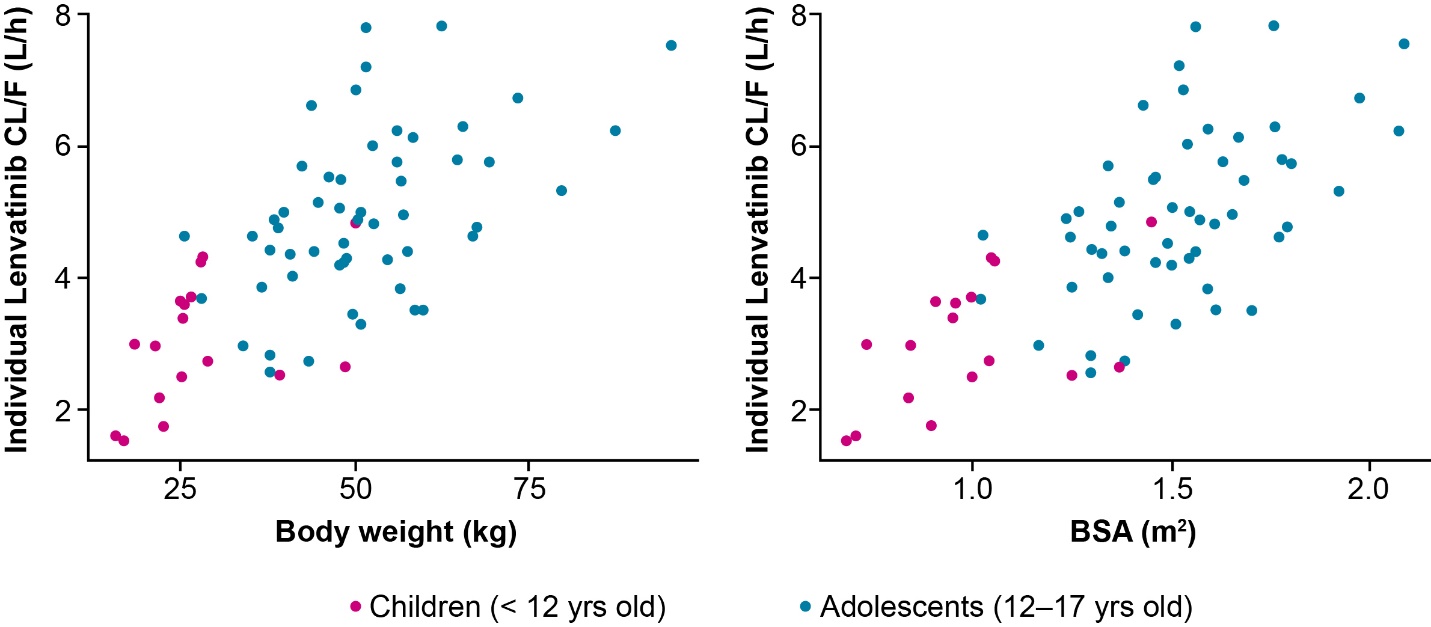


BSA, body surface area; CL/F, lenvatinib clearance.

**Supplementary References**

1. Doussau A, Asselain B, Le Deley MC, et al. Dose-finding designs in pediatric phase I clinical trials: comparison by simulations in a realistic timeline framework. *Contemp Clin Trials*. 2012;33(4):657-665.
2. Smith M, Bernstein M, Bleyer WA, et al. Conduct of phase I trials in children with cancer. *J Clin Oncol*. 1998;16(3):966-978.
3. Cheung YK. Sample size formulae for the Bayesian continual reassessment method. *Clin Trials*. 2013;10(6):852-861.
4. Lee DP, Skolnik JM, Adamson PC. Pediatric phase I trials in oncology: an analysis of study conduct efficiency. *J Clin Oncol*. 2005;23(33):8431-8441.
5. Cheung YK, Chappell R. Sequential designs for phase I clinical trials with late-onset toxicities. *Biometrics*. 2000;56(4):1177-1182.
6. Grande E, Díez JJ, Zafon C et al. Thyroid cancer: molecular aspects and new therapeutic strategies. J Thyroid Res 2012; 2012: 847108.
7. Matsuki M, Hoshi T, Yamamoto Y et al. Lenvatinib inhibits angiogenesis and tumor fibroblast growth factor signaling pathways in human hepatocellular carcinoma models. Cancer Med 2018; 7: 2641-2653.
8. Yakes FM, Chen J, Tan J et al. Cabozantinib (XL184), a novel MET and VEGFR2 inhibitor, simultaneously suppresses metastasis, angiogenesis, and tumor growth. Mol Cancer Ther 2011; 10: 2298-2308.
9. Roskoski R, Jr. Classification of small molecule protein kinase inhibitors based upon the structures of their drug-enzyme complexes. Pharmacol Res 2016; 103: 26-48.
10. Tian S, Quan H, Xie C et al. YN968D1 is a novel and selective inhibitor of vascular endothelial growth factor receptor-2 tyrosine kinase with potent activity in vitro and in vivo. Cancer Sci 2011; 102: 1374-1380.
11. Xie L, Xu J, Sun X et al. Apatinib for advanced osteosarcoma after failure of standard multimodal therapy: an open label phase II clinical trial. Oncologist 2019; 24: e542-e550.
12. Wilhelm SM, Dumas J, Adnane L et al. Regorafenib (BAY 73-4506): a new oral multikinase inhibitor of angiogenic, stromal and oncogenic receptor tyrosine kinases with potent preclinical antitumor activity. Int J Cancer 2011; 129: 245-255.
13. Matsui J, Yamamoto Y, Funahashi Y et al. E7080, a novel inhibitor that targets multiple kinases, has potent antitumor activities against stem cell factor producing human small cell lung cancer H146, based on angiogenesis inhibition. Int J Cancer 2008; 122: 664-671.
14. Grignani G, Palmerini E, Dileo P et al. A phase II trial of sorafenib in relapsed and unresectable high-grade osteosarcoma after failure of standard multimodal therapy: an Italian Sarcoma Group study. Ann Oncol 2012; 23: 508-516.
15. Duffaud F, Mir O, Boudou-Rouquette P et al. Efficacy and safety of regorafenib in adult patients with metastatic osteosarcoma: a non-comparative, randomised, double-blind, placebo-controlled, phase 2 study. Lancet Oncol 2019; 20: 120-133.
16. Davis LE, Bolejack V, Ryan CW et al. Randomized double-blind phase II study of regorafenib in patients with metastatic osteosarcoma. J Clin Oncol 2019; 37: 1424-1431.
17. Italiano A, Mir O, Mathoulin-Pelissier S et al. Cabozantinib in patients with advanced Ewing sarcoma or osteosarcoma (CABONE): a multicentre, single-arm, phase 2 trial. Lancet Oncol 2020; 21: 446-455.
